# *Lactobacillus crispatus* CCFM1339 Inhibits Vaginal Epithelial Barrier Injury Induced by *Gardnerella vaginalis* in Mice

**DOI:** 10.3390/biom14020240

**Published:** 2024-02-18

**Authors:** Xiaoyan Huang, Rumeng Lin, Bingyong Mao, Xin Tang, Jianxin Zhao, Qiuxiang Zhang, Shumao Cui

**Affiliations:** 1 State Key Laboratory of Food Science and Resources, Jiangnan University, Wuxi 214122, China; 6210112030@stu.jiangnan.edu.cn (X.H.); 6210113054@stu.jiangnan.edu.cn (R.L.); maobingyong@jiangnan.edu.cn (B.M.); xintang@jiangnan.edu.cn (X.T.); jxzhao@jiangnan.edu.cn (J.Z.); 2 School of Food Science and Technology, Jiangnan University, Wuxi 214122, China

**Keywords:** vagina epithelial barrier, *Lactobacillus crispatus*, BV, *Gardnerella vaginalis*

## Abstract

The vaginal epithelial barrier, which integrates mechanical, immune, chemical, and microbial defenses, is pivotal in safeguarding against external pathogens and upholding the vaginal microecological equilibrium. Although the widely used metronidazole effectively curtails *Gardnerella vaginalis*, a key pathogen in bacterial vaginosis, it falls short in restoring the vaginal barrier or reducing recurrence rates. Our prior research highlighted *Lactobacillus crispatus* CCFM1339, a vaginally derived *Lactobacillus* strain, for its capacity to modulate the vaginal epithelial barrier. In cellular models, *L. crispatus* CCFM1339 fortified the integrity of the cellular monolayer, augmented cellular migration, and facilitated repair. Remarkably, in animal models, *L. crispatus* CCFM1339 substantially abated the secretion of the barrier disruption biomarker E-cadherin (from 101.45 to 82.90 pg/mL) and increased the anti-inflammatory cytokine IL-10 (35.18% vs. the model), consequently mitigating vaginal inflammation in mice. Immunological assays in vaginal tissues elucidated increased secretory IgA levels (from 405.56 to 740.62 ng/mL) and curtailed *IL-17* gene expression. Moreover, *L. crispatus* CCFM1339 enhanced *Lactobacilli* abundance and attenuated *Enterobacterium* and *Enterococcus* within the vaginal microbiome, underscoring its potential in probiotic applications for vaginal barrier regulation.

## 1. Introduction

The human female vagina is a permeable canal, which is protected from the external environment by the vaginal epithelial barrier [1]. The female vaginal barrier primarily comprises four protective measures: mechanical, immune, microbial, and chemical barriers. The mechanical barrier, also known as the physical barrier, consists of an intact layer of vaginal epithelial cells connected through tight-junction and desmosomal proteins. The microbial barrier is predominantly governed by over 50 types of microorganisms, with *Lactobacilli* accounting for 70–80% of them [2]. The mucosa infiltrates the epithelial cells to enhance the mechanical capabilities of the vaginal barrier in collaboration with mucins [3,4,5]. Simultaneously, mucins work together with immunoglobulin G (IgG), immunoglobulin A (IgA), defensins, and secretory leukocyte protease inhibitor (SLPI) in the vaginal mucus to strengthen the immune barrier [4,6]. Closely intertwined with the other barriers, the chemical barrier consists of substances produced by vaginal bacteria such as lysozyme, lactic acid, and hydrogen peroxide. Alongside mucins, these chemicals disrupt the integrity of pathogenic microbial cell walls, reduce the vaginal pH, inhibit microbial growth, and closely monitor the host’s vaginal microecological balance [7,8]. These four components synergistically interact to form a cohesive barrier to prevent pathogenic bacteria or viruses from entering into the vaginal environment.

The integrity of the vaginal barrier can be compromised by various factors, including pathogenic invasion and unhealthy lifestyle habits such as poor hygiene practices. *Streptococcus agalactiae*, a common opportunistic pathogen and a dominant bacterium in aerobic vaginitis (AV), has the ability to stimulate shedding of vaginal epithelial cells by activating integrins and β-catenin signaling pathways, resulting in loss of barrier function and epithelial-mesenchymal transition [9]. *Gardnerella vaginalis* is the main cause of bacterial vaginitis (BV). By producing virulence factors like sialidase, prolidase, and putrescine, *G. vaginalis* can degrade mucin and other protective factors on the mucosal suface, thereby promoting shedding of epithelial cells [10,11]. Candidal vulvovaginitis (VVC) is a common fungal infection affecting the vagina. Under normal conditions, *Candida albicans* does not harm it; however, during its transition to the hyphal form, it actively penetrates host tissues by growing filaments, which disrupt epithelial cells and invade through the vaginal epithelial barrier [12]. There are also reports suggesting that HIV infection leads to disruption of skeleton proteins in vaginal epithelial cells, increased cell death rates, and the subsequent shedding of these cells [13].

Current international guidelines recommend the use of antibiotics, such as metronidazole and fluconazole, for the treatment of BV or VVC. However, these treatments have been associated with high rates of failure and recurrence [14]. This may be due to factors such as antibiotic resistance, an inability to eliminate polymicrobial biofilms, a failure to restore an acidic pH level, and an inability to facilitate *lactobacilli* dominance. Therefore, it is crucial to find an effective and safe treatment that can restore the vaginal microecology and repair the vaginal barrier. Probiotics are active microorganisms that naturally inhabit the vaginal environment and contribute to vaginal health by strengthening the vaginal barrier. Numerous in vivo, in vitro, and clinical studies have demonstrated that probiotics can effectively prevent vaginal infections by reducing the colonization of vaginal pathogens and inhibiting inflammation in the vagina. Heczko et al. [15] found that combining probiotics with an antimicrobial therapy for BV or VVC can effectively reduce recurrence rates. Ehrstrm et al. [16] discovered that short-term treatment with *lactobacillus* (5 days) followed by conventional BV or VVC treatment allowed *lactobacillus* colonization for up to 6 months while improving symptoms. Eric et al. observed that LACTIN-V intervention not only alleviated bacterial vaginitis but also reduced the release of IL-1α and E-cadherin biomarkers indicating epithelial barrier integrity [17].

In this study, our objective was to evaluate the efficacy of specific probiotic strains in enhancing cell barrier integrity through in vitro experiments. By comparing these strains with metronidazole’s effects on the vaginal epithelial barrier, we identified beneficial effects associated with *Lactobacillus crispatus* CCFM1339 on maintaining a healthy vaginal epithelial barrier. The finding from this study provided valuable insights into the potential application of *L. crispatus* CCFM1339 in the treatment of vaginitis.

## 2. Materials and Methods

### 2.1. Strain Preparation

The *L. crispatus* CCFM1339, FHNXY73M2, and *L. delbrueckii* DM8909 were obtained from the Culture Collection of Food Microorganisms (CCFM) of Jiangnan University. Isolated colonies were inoculated into MRS broth and cultured at 37 °C for 24 h. Subsequently, after three generations of exponential growth, the fermentation broths were subjected to high-pressure homogenization (800–1200 MPa) for 10 cycles to lyse the cells. The bacterial lysates were then filtered through 0.22 µm membranes and stored at −80 °C for subsequent cellular experiments.

For in vivo studies, the *lactobacilli* were centrifuged at 8000× *g* for 15 min at 4 °C, and the resulting pellets were washed twice with phosphate-buffered saline (PBS). The washed pellets were lyophilized and resuspended in PBS to a concentration of 10^11^ CFU/mL just prior to usage.

Additionally, *G. vaginalis* ATCC 14018, maintained in our laboratory, was cultivated in brain heart infusion (BHI) medium. The bacteria were subsequently centrifuged at 8000× *g* for 15 min at 4 °C, and the bacterial cells were washed three times with PBS. The cells were finally resuspended in PBS to a concentration of 10^10^ CFU/mL for use in animal experiments.

### 2.2. Cell Culture

Human epithelial cells VK2/E6E7 (ATCC CRL-2616) donated by Wuxi People’s Hospital were cultured in Dulbecco’s Modified Eagle Medium (DMEM, Gibco, Carlsbad, CA, USA) supplemented with 10% fetal bovine serum (FBS, Gibco, Carlsbad, CA, USA), and antibiotics (10 U/mL ampicillin, 10 µg/mL streptomycin) at 37 °C in a 5% CO_2_ humidified incubator. To establish human vaginal epithelial monolayer barrier model cells, the apical side (AP) of Transwell plates (Corning, polycarbonate membrane inserts, 0.4 mm pore size) was prewetted with chilled PBS solution. A suspension of 5 × 10^4^ VK2/E6E7 cells in 500 µL DMEM was added to each insert, followed by the addition of 1.5 mL medium to the basolateral side (BL). The plate was then incubated at 37 °C for approximately 21 days to allow the formation of a stable transepithelial electrical resistance (TEER)-based monolayer.

The experimental design included a control group, a model group, and treatment groups with *L. delbrueckii* DM8909 (DM8909), *L. crispatus* CCFM1339 (CCFM1339) and *L. crispatus* FHNXY73M2 (FHNXY73M2). Except for the control group, all other groups received DMEM containing 25 μg/mL lipopolysaccharide (LPS, *Escherichia coli* 055: B5, Sigma) for 24 h of intervention. Subsequently, the cells were washed with PBS and further treated with DMEM containing 5% (*v*/*v*) probiotic lysate for an additional 24 h [18].

### 2.3. Cell Epithelial Barrier Integrity Detection

Epithelial barrier integrity was assessed by following the established protocol [7]. Utilizing the Millicell-ERS Volt-Ohm Meter (Millipore, MA, USA), resistance measurements were conducted with electrodes oriented perpendicular to the monolayer cells and placed at the AP and BL poles of the cell filter membrane. Three independent measurements were performed for each well, and the mean TEER value was recorded. The resistance per unit area (Ω × cm^2^) was determined by multiplying the sample resistance by the membrane area [8].

### 2.4. Cell Permeability Analysis

Cell permeability was assessed as previously described by Zevin et al. [10]. Following TEER monitoring, the culture medium was removed, and the upper chamber was filled with phenol red-free DMEM medium (Gibco, USA) supplemented with fluorescein isothiocyanate-dextran 4 (FD-4, Sigma). Two hours post addition, the translocation of FD-4 from the upper insert to the lower chamber was measured using a fluorescence plate reader with excitation and emission wavelengths of 485 nm and 535 nm, respectively.

### 2.5. Scratch Assay

The scratch assay was conducted as previously outlined [19,20]. Initially, 5 × 10^4^ VK2/E6E7 cells were seeded into each well of a 6-well culture plate in 2 mL of DMEM medium, and the plates were incubated at 37 °C with 5% CO_2_ for 24 h. Subsequently, a liner scratch was created across the well using a sterile P200 pipette tip to disrupt a single cell layer. The wells were then rinsed with PBS and supplemented with DMEM containing 5% bacterial lysate (*v*/*v*). Images were recorded at five predetermined reference points per well and analyzed using the ImageJ software at both 0 and 24 h. The scratch healing rate was calculated using the formula: scratch healing rate (%) = (W_0_ − W_24_)/W_0_ × 100%, where W_0_ represents the initial scratch area and W_24_ represents the scratch area at 24 h.

### 2.6. Animal Experiment

Seven-week-old female BALB/c mice, acquired from Beijing Vital River Laboratory Animal Technology Co., Ltd. (Beijing, China), were housed in wire cages under conditions of 20–22 °C, 50 ± 10% humidity, and a 12-h light/dark cycle. The conduct of this study was overseen by the Institutional Animal Care and Use of Committee at Jiangsu Institute of Parasitic Diseases (IACUC-JIPD-2023111).

**Figure 1:**
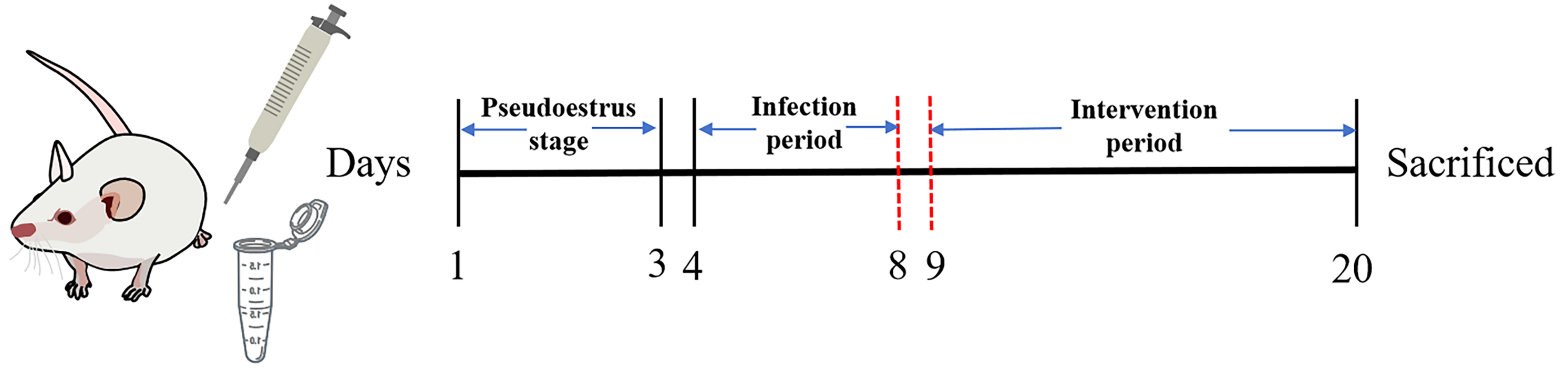
Animal experimental design time flow diagram.

Thirty-six mice were randomly allocated to 6 groups as follows: control group (Control); model group (Model); metronidazole-treated group (Metronidazole); *L. delbrueckii* DM8909 group (DM8909); *L. crispatus* CCFM1339 group (CCFM1339); and *L.* FHNXY73M2 group (FHNXY73M2). All mice were provided with a standard commercial diet and allowed free access to water. The BV model was established according to the methods described previously [21]. Three days prior to infection (on days 1–3), all mice except those in the control group received a subcutaneous injection of 100 µL of estradiol benzoate (0.5 mg dissolved in 100 µL sesame oil). On day 4, vaginal inoculation was performed with 20 µL of *G. vaginalis*, which was repeated daily for five consecutive days up to day 8. The specific inoculation procedure involved inserting the pipette tip approximately 5 mm into the vagina. Following inoculation, mice were inverted for 1–2 min to prevent leakage. From day 9 onward, the experimental mice received a daily vaginal inoculation of 20 µL of *Lactobacillus* for a total of 12 days. The metronidazole group was treated with a solution of 1.62 mg per 20 g of mouse body weight dissolved in PBS. Mice in the control group were intravaginally inoculated with 20 µL of PBS once daily. Vaginal lavage samples were collected on day 19, after which all experimental mice were sacrificed on day 20. The schedule for the animal experiment is presented in Figure 1.

### 2.7. Histopathological Observation

The mice were euthanized and sacrificed at the end of the experiment. The vaginal tissue was carefully excised and preserved using 4% paraformaldehyde fixation, subsequent embedding in paraffin, and then sectioned into 5 mm thick slices. These sections were stained with hematoxylin and eosin (H&E) for histological examination. The stained vaginal tissue samples were analyzed using a Panoramic MIDI scanner (3DHistech Ltd., Budapest, Hungary) for microscopic visualization.

### 2.8. ELISA

Vaginal tissue samples (20 mg) were placed in 200 µL of Radioimmunoprecipitation assay (RIPA) buffer containing 2% (*v*/*v*) protease inhibitor cocktail and 2% (*v*/*v*) phosphatase inhibitor mixture (SenBeiJia, Nanjing, China). The tissue samples were then homogenized using a high-throughput tissue grinder at a frequency of 65 Hz for 45 s per cycle, totaling 8 cycles. Subsequently, the homogenates were centrifuged at 3000 rpm for 15 min at 4 °C. The supernatants were carefully collected for the resulting vaginal tissue and were utilized for the quantification of interleukin-1β (IL-1β), tumour necrosis factor α (TNF-α), myeloperoxidase (MPO), interleukin-10 (IL-10), soluble E-cadherin (sECAD), zonula occludens protein 1 (ZO-1), claudin-1 (CLDN1), occludin (OCLN), secretory immunoglobulin A (sIgA), immunoglobulin G (IgG), and Hydrolase B Domain 2 (HBD-2) levels in accordance with the manufacturer’s instructions provided within the assay kit (SenBeiJia, China).

### 2.9. cDNA Generation and qPCR

The total RNA of mouse vaginal tissues was extracted using the trizol method [22]. Subsequently, a commercial reverse transcription kit (Vazyme, Nanjing, China) was used to convert total RNA into complementary DNA (cDNA). The ChamQ Universal SYBR qPCR Master Mix (Vazyme, China) was employed to configure the fluorescence quantitative PCR reaction system (10 µL), composed of 5 µL of SYBR qPCR Master Mix, 0.5 µL of upstream primers, 0.5 µL of downstream primers, 3 µL of ddH_2_O, and 1 µL of DNA template. The RT-qPCR reaction program was as follows: 95 °C for 30 s, 40 cycles (95 °C for 30 s; 60 °C for 30 s), and a dissolution curve from 65 °C to 95 °C, incremented by 0.5 °C/s. *GAPDH* was used as the internal reference gene, and the target gene expression level was determined based on the 2^−ΔΔCt^method [23]. The primers applied in this study are detailed in Table 1.

### 2.10. Vaginal Microbiota Analysis

On day 19, the vaginal lavage fluid was collected by gently irrigating the mice with 350 µL of sterile PBS, administered in 7 aliquots of 50 µL each. The collected fluid was then transferred into a sterile 1.5 mL tube for further analysis. DNA extraction from the vaginal fluid samples was performed using the Fast DNA^TM^ Spin Kit for soil (MP Biomedicals, Santa Ana, CA, USA). PCR was performed with the primers 338F (5′-CCTAYGGGRBGCASCAG-3′) and 806R (5′-GGACTACNNGGGTATCTAAT-3′) to amplify the V3–V4 region of the 16S rRNA gene. The PCR reaction system and reaction conditions were adapted from those described by Mitchell et al. [24]. The amplified fragments were purified, and the DNA concentration (ng/µL) was determined using a Nano Drop Spectrophotometer (Thermo Scientific, Waltham, MA, USA). Library construction and high-throughput sequencing were carried out on the Illumina HiSeq PE250 platform by Tianhao Biotechnology Company, Ltd. (Shanghai, China). The raw data were processed using QIIME (v.2.0.0), with sequences clustered into operational taxonomic units (OTUs) based on a similarity exceeding 97%. The diversity analysis of the vaginal microbiota was conducted in R language, and significant taxonomic differences between groups were assessed using STAMP (version 2.1.3).

### 2.11. Statistical Analysis

Data were analyzed and a graphical representation was created using GraphPad Prism version 9.0. For multiple group comparisons, one-way analysis of variance (one-way ANOVA) or repeated measures ANOVA was employed, followed by Dunnett and Sidak post hoc tests to assess differences between and within groups. Statistical significance was determined at *p* < 0.05. Data are presented as mean ± standard deviation.

## 3. Results

### 3.1. Screening for Lactobacillus Strains Capable of Enhancing the Vaginal Epithelial Barrier

**Figure 2:**
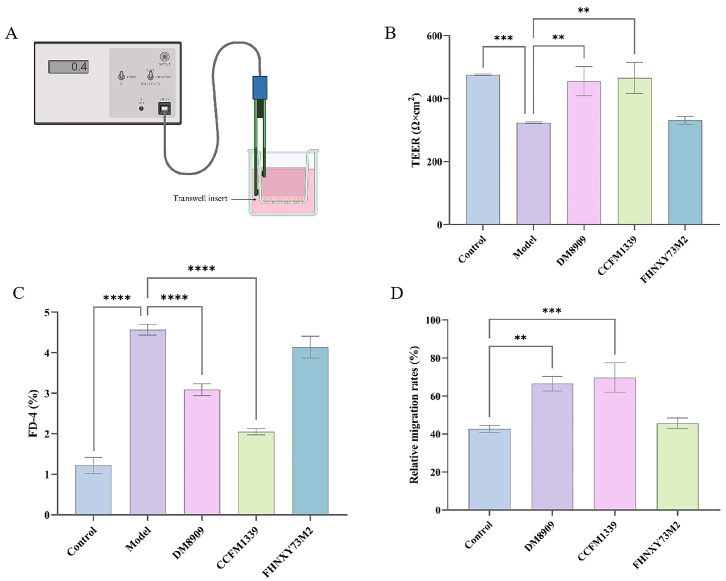
The regulation of VK2/E6E7 monolayer barrier cells by Lactobacillus strains. (**A**) The Transwell model of the vaginal epithelium; (**B**) transepithelial electrical resistance; (**C**) the permeability of FD-4; (**D**) the wound area change treated at 24 h after the scratch; ** *p* < 0.01, *** *p* < 0.001, **** *p* < 0.0001 vs. model group.

Our initial findings suggested that *L. crispatus* CCFM1339 was selected due to its ability to preserve the integrity of vaginal epithelium monolayers, decrease permeability, and promote cell migration. *L. crispatus* FHNXY73M2, another strain, showed negative results in these aspects and was used as a control. *L. delbrueckii* DM8909, a commercial probiotic for BV treatment, served as an additional control. The Transwell model of the vaginal epithelium is shown in Figure 2A. Barrier disruption post LPS treatment was observed, as evidenced by reduced the TEER and increased permeability (Figure 2B–D). Treatment with *L. crispatus* CCFM1339 following LPS exposure resulted in a TEER recovery of over 44.02% relative to the model group, coupled with a significant reduction in FD-4 permeability (*p* < 0.0001). Moreover, the scratch assay showed that *L. crispatus* CCFM1339 enhanced wound healing rates by 63%. *L. delbrueckii* DM8909 exhibited comparable effects on the integrity and permeability to *L. crispatus* CCFM1339. Concurrently, *L. delbrueckii* DM8909 demonstrated a significant stimulatory effect on cell migration when contrasted with the control group, yielding an augmentation of 55.53%, which was marginally less efficacious than that observed with *L. crispatus* CCFM1339. In contrast, *L. crispatus* FHNXY73M2 exhibited no significant advantages over the model group in any of these parameters.

### 3.2. Effects of L. crispatus CCFM1339 on Mechanical Barrier

**Figure 3:**
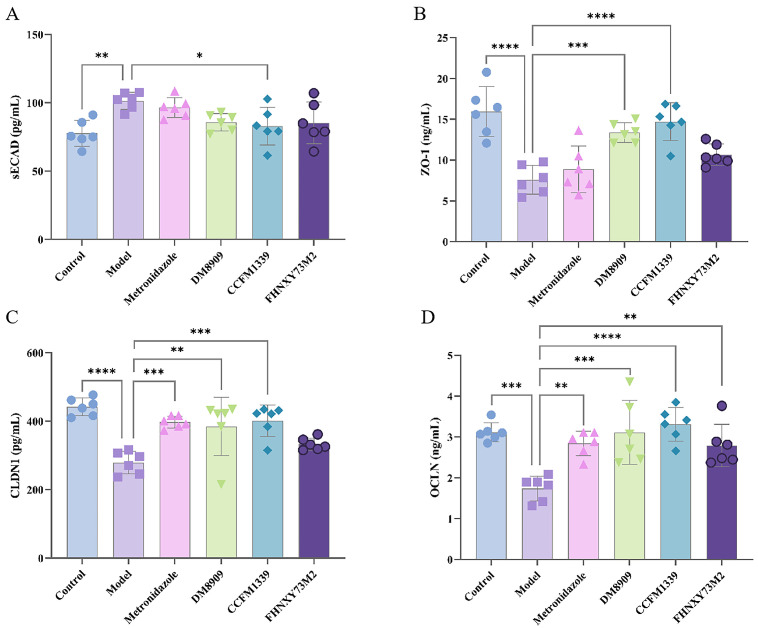
Mechanical protein expression in vaginal tissue: (**A**) sECAD; (**B**) ZO-1; (**C**) CLDN1; (**D**) OCLN; * *p* < 0.05, ** *p* < 0.01, *** *p* < 0.001, **** *p* < 0.0001 vs. model group.

E-cadherin, a pivotal component of adherens junctions in epithelial cells, is liberated when epithelial barriers experience weakened intercellullar adhesion [17]. As depicted in Figure 3A, infection with *G. vaginalis* markedly diminished soluble E-cadherin levels in mice (from 101.45 to 82.90 pg/mL), potentially due to the action of vaginolysin and sialic acid secreted by *G. vaginalis*, which degrade vaginal mucus and compromise the vaginal epithelial barrier [25]. The use of metronidazole, *L. delbrueckii* 8909, and *L. crispatus* FHNXY73M2 failed to revert soluble E-cadherin levels. Only *L. crispatus* CCFM1339 administration elicited a significant recovery in E-cadherin expression (*p* < 0.05).

ZO-1, CLDN1, and OCLN proteins are standard markers for assessing barrier integrity [8,26,27]. Abundant tight junctions are present between basal cells of the vaginal epithelium, with tight-junction proteins predominantly localized in the lower two-thirds of the epithelium, forming a spiderweb-like pattern, indicative of their role in cell adhesion [28]. Model group vaginal tissues exhibited a significant decline in tight-junction protein expression versus controls (*p* < 0.001). Metronidazole therapy partially restored CLDN1 (Figure 3C) and OCLN proteins (Figure 3D), yet failed to affect ZO-1 expression (Figure 3B). In contrast, the CCFM1339 group mice displayed a marked increase in tight-junction protein expression, approaching control levels. *L. delbrueckii* DM8909 exhibited a trend similar to *L. crispatus* CCFM1339, although the effects were less pronounced. The control *L. crispatus* FHNXY73M2 strain, with the exception of OCLN, showed no significant effects on other indicators.

### 3.3. Effects of L. crispatus CCFM1339 on Inflammatory Barrier

The proinflammatory cytokine IL-1β acts synergistically in the inflammatory cascade, potentiating the host’s immune response and compromising barrier integrity as evidenced in reference [29]. TNF-α is a key gene expression in the activation of the downstream NF-κB pathway, serving as a biomarker for NF-κB activation [30]. Myeloperoxidase (MPO), predominantly expressed in neutrophils within inflamed tissues, serves as a proxy for neutrophil aggregation in vaginal tissues [31].

**Figure 4:**
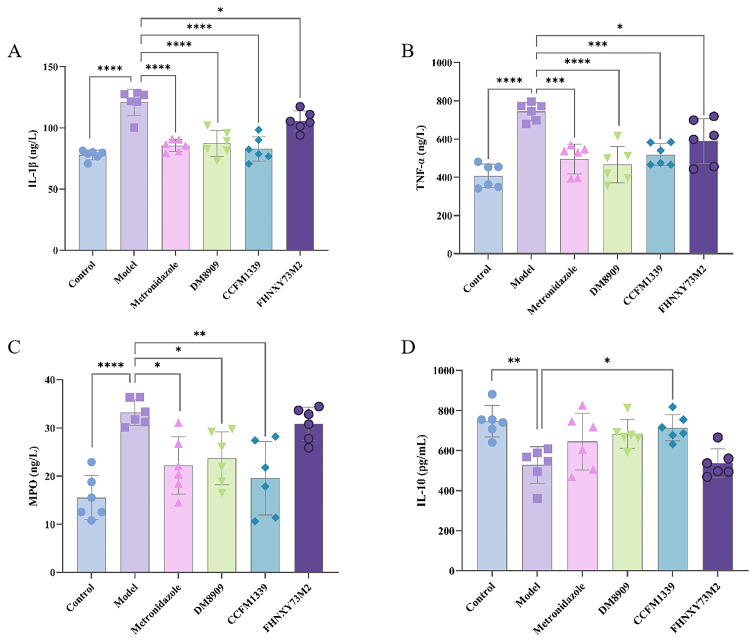
Changes in inflammatory factors in vaginal tissue. (**A**) IL-1β; (**B**) TNF-α; (**C**) MPO; (**D**) IL-10; * *p* < 0.05, ** *p* < 0.01, *** *p* < 0.001, **** *p* < 0.0001 vs. model group.

To assess vaginal inflammation subsequent to *G. vaginalis* infection, levels of these immune factors within the vaginal tissue were quantified. As depicted in Figure 4A, the IL-1β content noticeably rose from 79.84 to 127.44 ng/L in the model group, and this elevation was reduced to 90.56 ng/L. following the metronidazole treatment. The DM8909 and FHNXY73M2 groups exhibited mean IL-1β levels of 95.75 and 94.03 ng/L, respectively, while the CCFM1339 intervention yielded an outcome of 90.05 ng/L (*p* < 0.0001). All experimental groups demonstrated a reduction in IL-1β secretion, with metronidazole and CCFM1339 demonstrating the most pronounce effects. A similar pattern was evident for TNF-α levels (Figure 4B), with the CCFM1339 group showing the most significant decrease of 31.27% (*p* = 0.0004). MPO expression was also detected (Figure 4C). In the control group, MPO expression was significantly elevated compared to controls (*p* = 0.0011), suggesting *G. vaginalis*-induced neutrophil infiltration into vaginal tissues. The FHNXY73M2 exhibited a relatively faint inhibitory influence on MPO activity. The vaginal administration of metronidazole and DM8909 suppressed MPO levels to 22.22 and 23.68 ng/L, respectively (*p* = 0.0119; *p* = 0.0386). CCFM1339 demonstrated the most potent inhibitory action on this enzyme, reaching an expression level of 19.54 ng/L (*p* = 0.0011).

IL-10 stands as a pivotal anti-inflammatory cytokine, produced by a spectrum of cell types including lymphocytes, macrophages, and other immune cells [32]. As delineated in Figure 4D, the secretion of IL-10 from the model group was determined to be 528.22 pg/mL, marking a significant decrease in comparison to the control group’s level of 746.84 pg/mL (*p* = 0.0028). Conversely, the IL-10 content within the CCFM1339 treated group exhibited a notable enhancement of 35.18% (*p* = 0.0145), surpassing that of metronidazole (22.15%), DM8909 (29.51%), and FHNXY73M2 (1.73%). These findings corroborated that CCFM1339 exerts a regulatory influence on alleviating vagina inflammation.

### 3.4. Effects of L. crispatus CCFM1339 on Anti-Infection Index

**Figure 5:**
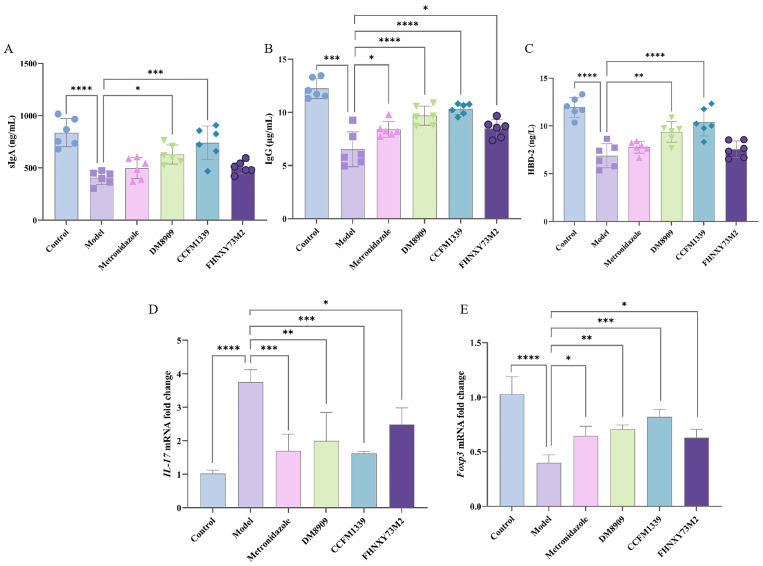
Effects of *L. crispatus* CCFM1339 on the vaginal immune barrier indicators. (**A**) sIgA; (**B**) IgG; (**C**) HBD-2; (**D**) *IL-17* gene; (**E**) *Foxp3* gene; * *p* < 0.05, ** *p* < 0.01, *** *p* < 0.001, **** *p* < 0.0001 vs. model group.

In the context of mucosal immunity, IgA antibodies, with a particular emphasis on sIgA, represent the paramount first line of defense mechanism orchestrated by mucosal epithelial cells to counteract HIV-1 infection and other pathogenic threats [33,34]. As depicted in Figure 5A, the determination of sIgA content highlighted a notable reduction in the vaginal tissue of the experimental subjects by day 19, plummeting from an initial 836.87 to 405.56 ng/mL (*p* < 0.0001). Notably, the interventions involving metronidazole and FHNXY73M2 did not significantly increase sIgA secretion (*p* > 0.05). The DM8909 treatment group recorded an sIgA level of 628.51 ng/mL (*p* = 0.014), which was found to be lower compared to the CCFM1339 group, demonstrating a level of (740.62 ng/mL, *p* = 0.0001). IgG, the predominant immunoglobulin presents in cervical and vaginal secretions, functions by impeding the adherence of microorganisms to epithelial surfaces, thereby facilitating the neutralization and clearance of pathogens and their toxins within the mucosal milieu, exerting a pivotal anti-infective role [35]. We further assessed the IgG content within the vaginal tissues, as illustrated in Figure 5B. The model group’s IgG content was substantially augmented by 58.14% following CCFM1339 intervention (*p* < 0.0001), a response markedly superior to those observed with metronidazole, DM8909, and FHNXY73M2, which resulted in increments of 28.80%, 48.62%, and 29.81%, respectively.

Additionally, we assessed the levels of HBD-2 in vaginal tissues, as presented in Figure 5C. HBD constitutes a class of low-molecular-weight peptides exhibiting broad-spectrum, potent antibacterial activity within various compartments, including the skin, mucosal epithelia, and vaginal tract. It has been demonstrated that HBD peptides contribute to the defense against infections in the female reproductive tract, functioning as a vital component in the formation of a vaginal barrier [36]. In comparison to the model group, FHNXY73M2 did not significantly augment the secretion of HBD-2. Conversely, CCFM1339 enhanced HBD-2 secretion, outperforming DM8909, and approached the levels observed in the control group. This finding corroborates earlier reports suggesting that *Lactobacillus* can interact bidirectionally with vaginal epithelial cells, potentiating the secretion of antimicrobial peptides, facilitating their lysis, and enabling their uptake as a nitrogen source, thereby ameliorating the balance of the vaginal microecology [37].

*IL-17*-mediated immunity response plays a critical role in the host defense mechanism of the mouse vagina [38]. *Foxp3*, a pivotal transcriptional regulator of regulatory T cells (Tregs), collaborates with nuclear factors of activated T cells to form a synergistic complex, leading to the upregulation of genes that suppress treg activity. Research indicates a positive correlation between *Foxp3* expression and the inhibitory function of Tregs [39,40]. As depicted in Figure 5D,E, *L. crispatus* CCFM1339 markedly inhibited the expression of the *IL-17* gene while enhancing *Foxp3* expression, exhibiting a superior effect compared to metronidazole, *L. delbrueckii* DM8909, and FHNXY73M2 in terms of gene expression modulation. These findings suggest that *L. crispatus* CCFM1339 can contribute to the adaptive immune defense of the mouse vaginal barrier, thereby enhancing the efficacy of the vaginal immune barrier.

### 3.5. Histopathological Analysis

**Figure 6:**
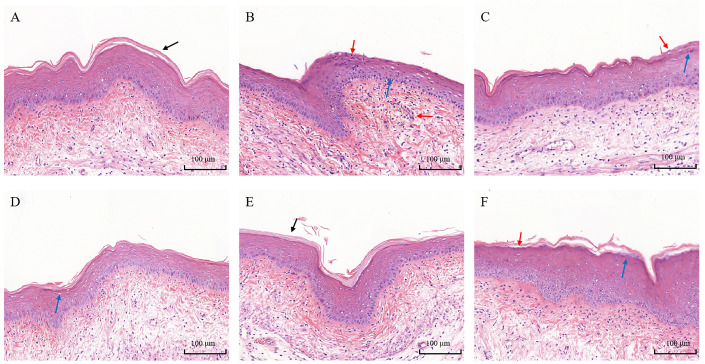
Pathological status of vaginal tissue. (**A**) Control; (**B**) model; (**C**) metronidazole; (**D**) DM8909; (**E**) CCFM1339; (**F**) FHNXY73M2; black arrow—smooth vaginal epithelium; blue arrow—local inflammatory cell infiltration of vaginal mucosa; red arrow—superficial erosion and holes.

Through the implementation of hematoxylin and eosin (H&E) staining on mouse vaginal tissues, a comprehensive assessment of the integrity and inflammatory status of the vaginal barrier in mice can be achieved. In the control group, the mouse vaginal mucosa exhibited integrity, featuring a discernible keratinized layer on its surface (Figure 6A). Conversely, in the model group, the keratinized layer was absent, with an observed proliferation of squamous epithelial cells and a conspicuous infiltration of inflammatory cells within the mucosal layer (Figure 6B). Treatment with metronidazole elicited a recovery response in the mice, suggesting the presence of an endogenous repair mechanism activated post pathogen clearance (Figure 6C). *L. delbrueckii* DM8909 demonstrated a reduction in inflammatory cell infiltration; however, there was a faint manifestation of epithelial erosion on the vaginal mucus (Figure 6D). The *L. crispatus* CCFM1339 group exhibited a restored, progressively continuous epithelial layer, diminished interstitial edema, a marked decrease in inflammatory cell infiltration, and the re-emergence of a keratinized layer on the mucosal surface. Those outcomes suggest that *L. crispatus* CCFM1339 is instrumental in promoting the repair of vaginal mucosa, alleviating inflammatory symptoms within vaginal tissue, and enhancing the structural integrity of the mouse vaginal tract (Figure 6E). Although the *L. crispatus* FHNXY73M2 group demonstrated improved epithelial continuity, there was partial congestion in the vaginal interstitium and slight edema in the connective tissue area (Figure 6F).

### 3.6. Microbiological Analysis of Mouse Vagina

**Figure 7:**
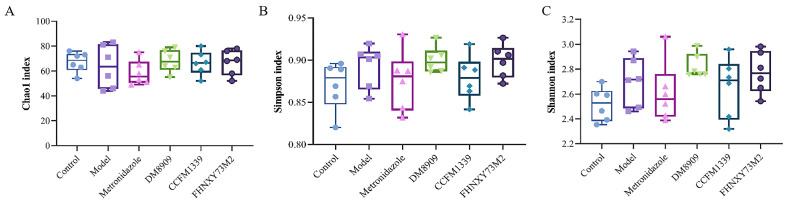
Analysis of alpha diversity. (**A**) Chao1 index; (**B**) Simpson index; (**C**) Shannon index.

The maintenance of a balanced vaginal microbiota is crucial for the health and integrity of the vaginal barrier. The proliferation of *G. vaginalis* can disrupt the equilibrium of the vaginal microecology, leading to disturbances in the vaginal microbiota. Research has highlighted the importance of appropriate probiotic administration in sustaining a healthy vaginal microbiota and in the prevention or treatment of vaginal infectious diseases [41]. To investigate the alterations in the overall structure of the vaginal microbiota among various intervention groups, we utilized α-diversity indices (Chao1, Shannon, and Simpson) to assess the changes in species evenness and richness in mice infected with *G. vaginalis* following intervention with metronidazole, DM8909, CCFM1339, and FHNXY73M2. As shown in Figure 7, the infection with vaginal *G. vaginalis* resulted in a decrease in the richness of the mouse vaginal microbiota, as indicated by the Chao1 index, with a slight increase in evenness, as reflected by the Simpson and Shannon indices, although these changes were not statistically significant (*p* > 0.05). In comparison to the model group, the metronidazole group exhibited reduced evenness and richness of the microbiota, as measured by the Chao1, Simpson, and Shannon indices. The intervention groups involving DM8909, CCFM1339, and FHNXY73M2 demonstrated an increase in the Chao1 index. Simultaneously, the Simpson index for metronidazole exhibited a negligible difference when compared to CCFM1339, whereas the decline in the Shannon index was significantly more substantial in the metronidazole group compared to CCFM1339. This is likely due to the broad-spectrum bactericidal activity of metronidazole, which led to a reduction in the variety of microbial species and a decrease in the richness within the murine vaginal microbiota. In contrast, the decreased Simpson and Shannon indices observed in the CCFM1339 group may be attributed to the subsequent dominance of *lactobacillus* in the microbiota following its intervention, which altered the evenness of the murine vaginal microbiota and fostered a shift towards a balanced and stable state.

**Figure 8:**
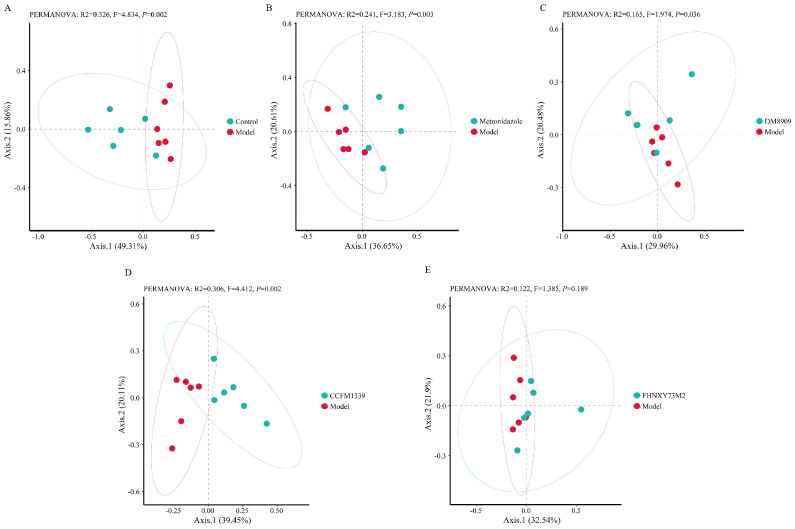
Analysis of beta diversity. (**A**) Control vs. model; (**B**) metronidazole vs. model; (**C**) DM8909 vs. model; (**D**) CCFM1339 vs. model; (**E**) FHNXY73M2 vs. model.

β−diversity is a measure that encapsulates the spatial distance and dissimilarity among groups, as well as the heterogeneity within biological communities. To assess the alterations in the comprehensive composition of samples following an intervention, we employed the unweighted Bray−Curtis index as a measure of dissimilarity within the framework of permutational multivariate analysis of variance (PERMANOVA). As depicted in Figure 8, a statistically significant disparity in species composition was observed between the control group and the model group. Notably, the core microbiota of the model group exhibited a substantial shift. The metronidazole, DM8909, and FHNXY73M2 groups were found to be situated closer to the core region of the model group, without demonstrating clear separation. Conversely, the CCFM1339 group exhibited a partial deviation from the cluster of the model group, suggesting a potential beneficial effect of CCFM1339 in enhancing the compositional integrity of the vaginal microbiota.

**Figure 9:**
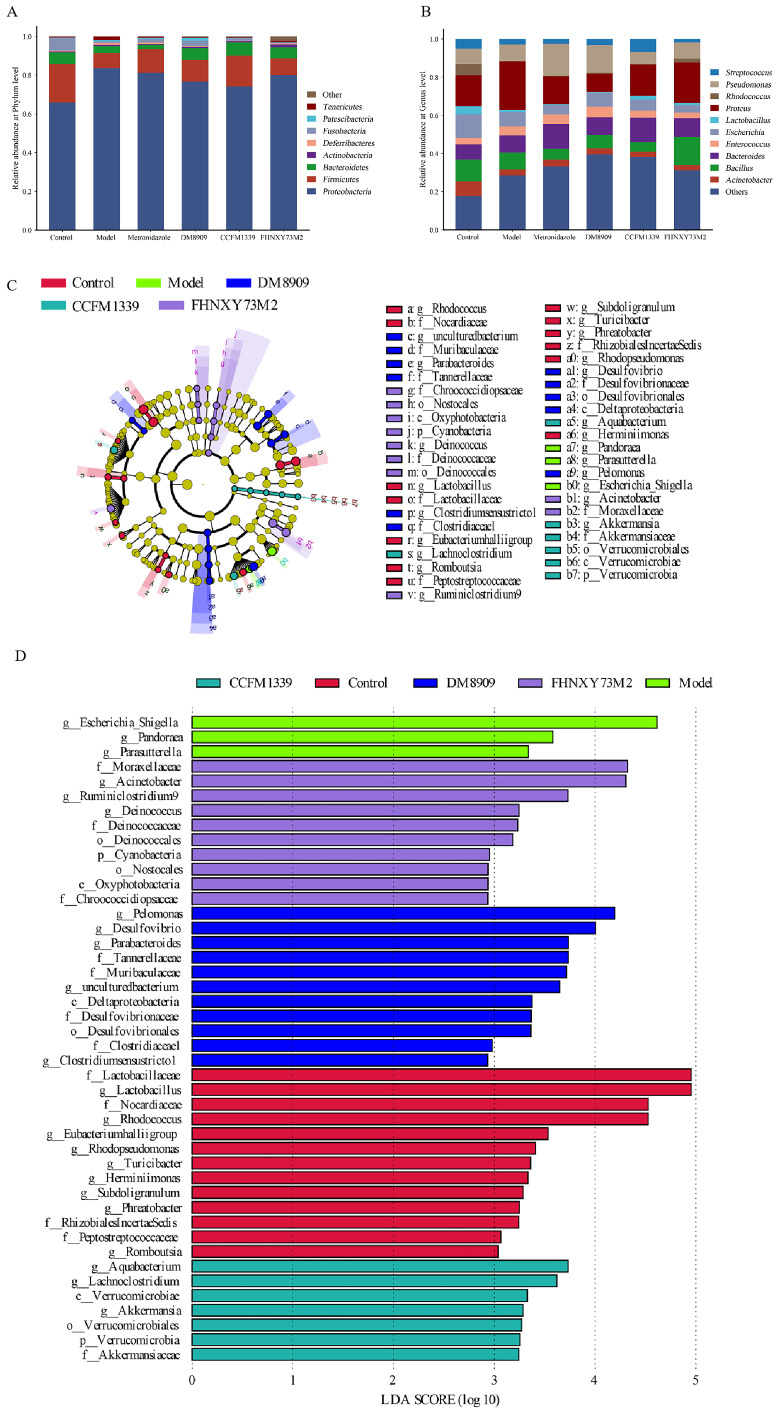
Analysis of different bacterial genera of vaginal microbiota. (**A**) Relative abundance of the vaginal microbiota at phylum level; (**B**) relative abundance of the vaginal microbiota at genus level; (**C**) branching map of species evolution; (**D**) histogram of LDA value distribution.

To investigate the composition of microbial communities across various samples and the structural transformations subsequent to intervention, we conducted a comparative analysis of the constituent species and their relative abundance in stacked bar charts, focusing on the phylum and genus levels. Figure 9A illustrates that the vaginal microbiota of all mouse groups predominantly comprised *Proteobacteria*, *Firmicutes*, and *Bacteroidetes*. Notably, the CCFM1339 group exhibited a significant decrease in *Proteobacteria* relative abundance and an associated increase in *Firmicutes* compared to the model group. Similarly, the relative abundance of *Proteobacteria* was also attenuated in the metronidazole, DM8909, and FHNXY73M2 groups relative to the model group. At the genus level, the compositional dominance was observed among genera such as *Escherichia-Shigella*, *Bacillus*, *Lactobacillus*, and *Streptococcus*. As depicted in Figure 9B, the model group demonstrated a marked enhancement in *Escherichia-Shigella* abundance (from 16.40% to 25.64%) and a concomitant reduction in *Lactobacillus* (from 4.10% to 1.06%) when compared to the control group. The administration of CCFM1339 reversed these trends, promoting an increase in *Lactobacillus* abundance while concurrently diminishing the relative proportions of *Pseudomonas* and *Escherichia-Shigella*. When considering the alterations in bacterial abundance at both the phylum and genus levels, CCFM1339 was found to exert regulatory effects akin to those of a probiotic, fostering the establishment of symbiotic relationships with health-promoting bacteria and countering the proliferation of pathogenic vaginal microorganisms.

To delineate the nuanced discrepancies in the microbial diversity and community architecture, linear discriminant analysis effect size (LEfSe) was employed to pinpoint the genera that exhibited differential abundance across the respective groups. Figure 9C,D elucidate that the metronidazole group lacked significant variation in genera. The model group, in contrast, harbored a conspicuously higher abundance of *Escherichia-Shigella*, whereas the control group demonstrated a significant elevated presence of *Lactobacillus*. Within the DM8909 group, the genera *Desulfovibrio* and *Pseudomonas* maintained their elevated status post intervention. Conversely, in the CCFM1339 group, *Akkermansia*, vested in *Firmicutes*, was found to be significantly more abundant following intervention, suggesting a pivotal role in the modulation of microbial community composition.

## 4. Discussion

Vaginal tract infections represent a prevalent category of microbial disorders globally. Owing to the distinctive anatomical features of the female reproductive tract and the fluctuating nature of the menstrual cycle, the female reproductive system is particularly susceptible to injury inflicted by invasive pathogenic bacteria, culminating in the compromise of the vaginal epithelial barrier [42]. In this study, we employed *G. vaginalis* to infect the vaginas of mice, thereby establishing a model of vaginal barrier disruption that was previously characterized [43]. Subsequently, we assessed the restorative properties of *L. crispatus* CCFM1339 on the vaginal barrier by examining the mechanical, immune, and microbial components of this barrier. Our findings indicate that *L. crispatus* CCFM1339 exerted a superior regulatory effect on the vaginal epithelial barrier when compared to metronidazole and *L. delbrueckii* DM8909 and *L. crispatus* FHNXY73M2. This beneficial effect may be attributed to the upregulation of tight-junction proteins in vaginal tissue, the downregulation of inflammatory factor expression, and the enhancement of *Lactobacillus* abundance. These mechanisms collectively underscore the potential of *L. crispatus* CCFM1339 as a therapeutic agent for restoring and maintaining vaginal health.

In female vaginal mucosal epithelial cells, cell adhesions are tightly linked via adherens junctions. This arrangement facilitates the targeted transport of epithelial secretions by isolating the basement membrane from the apical free end, thereby preventing the movement of small molecular substances between cells and further inhibiting the damage caused by pathogenic microbes to the basal cells of the vaginal epithelium [44]. In the current investigation, it was observed that *L. crispatus* CCFM1339 mitigated the release of E-cadherin in vaginal tissues and augmented the expression of tight-junction proteins. In contravention, metronidazole failed to reduce the secretion of E-cadherin, indicating that antibiotics may hinder pathogenic bacteria; however, they do not remediate the vaginal barrier (Figure 5). This outcome is presumably attributable to the barrier-strengthening effects of lactic acid produced by CCFM1339. A prior study showed that lactic acid within the vaginal microenvironment catalyses the upregulation of tight-junction proteins in vaginal epithelial cells, consequently reinforcing the integrity of the vaginal epithelial barrier [8]. On the converse side, *G. vaginalis* markedly elevates epithelial cell mortality in a cell-specific manner, compromising the integrity of the epithelial barrier [45].

Prolonged and excessive immune responses have been identified as a significant risk factor for the compromise of the vaginal barrier. Clinical studies have substantiated a strong correlation between *G. vaginalis* infection and the dysregulation of the vaginal immune environment [46]. IL-1β, a critical mediator of the mucosal barrier and a potential biomarker for vaginal inflammation, has been demonstrated to activate secondary inflammatory responses [47]. Hedge and colleagues [48] reported that women exhibiting a higher morphotype diversity of *G. vaginalis* in vaginal smears had increased levels of IL-1β in their vaginal secretions. In alignment with these observations, chronic infection with *G. vaginalis* was found to induce a significant increase in the secretion of IL-1β in vaginal tissues (Figure 6), with corresponding elevations in the levels of TNF-α and MPO within the vaginal tissues, compared to the control group. Histological sections of the vagina (Figure 7) revealed inflammatory cell infiltration, alterations in epithelial continuity, and mild erosion due to *G. vaginalis* infection. Antibiotic and probiotic interventions were found to significantly reduce the expression of proinflammatory factors in vaginal tissues; however, only *L. crispatus* CCFM1339 demonstrated a significant capacity to enhance the secretion of the anti-inflammatory cytokine IL-10 in vaginal tissues. While both IL-1β and TNF-α levels decreased in the groups treated with *L. delbrueckii* DM8909 and *L. crispatus* FHNXY73M2, IL-10 levels did not show a significant change. It is hypothesized that *L. crispatus* CCFM1339 may mitigate vaginal inflammation by orchestrating the balance between proinflammatory and anti-inflammatory factors, diminishing inflammatory cell infiltration, and fostering the repair of the epithelial barrier.

IgG constitutes the predominant immunoglobulin class within type II vaginal mucosa. It is produced by circulating plasma cells and is transported to the reproductive tract epithelium through the Fc receptor on vaginal epithelial cells [49]. Although IgA and IgM are also present in the vaginal mucosa, their levels are notably lower than those of IgG. IgG plays a pivotal role in coordinating functional activities such as opsonization, neutralization, complement fixation, the inhibition of epithelial phagocytosis or cell transfer, and antibody-dependent cellular cytotoxicity [50,51]. Although IgA and IgM are also present in the vaginal mucosa, their levels are notably lower than those of IgG [52]. In our investigation, we observed that persistent infection with *G. vaginalis* led to reduced levels of vaginal IgG, sIgA, and HBD-2, with the *L. crispatus* CCFM1339 demonstrating significantly higher expression levels than both the control model and metronidazole-treated groups (*p* < 0.05). Despite metronidazole treatment significantly lowering inflammatory factors, it failed to appreciably elevate the levels of vaginal immunoglobulins. In contrast, CCFM1339 not only exerted an anti-inflammatory effect but also enhanced the expression of antimicrobial peptides in mucosal tissues, providing empirical support for the synergistic enhancement of probiotic and antibiotic effects. Furthermore, we assessed the influence of CCFM1339 on vaginal adaptive immune factors, including IL-17 and Foxp3. Increasing evidence indicates that IL-17A and IL-17 play a role in host defense within the mouse vagina. For example, mice deficient in IL-17A or IL-17 receptor A (IL-17RA) exhibit increased susceptibility to *Neisseria* infection [53]. In an estrogen-induced VVC mouse model, mice produced abundant IL-17A upon *Candida albicans* assault and exhibited significant neutrophil infiltration into the vagina [54]. In our estrogen-induced bacterial vaginosis model, substantial expression of vaginal IL-17 mRNA was detected, suggesting the induction of adaptive immunity by *G. vaginalis.* CCFM1339 intervention effectively reduced the expression of both *IL-17* and *Foxp3* genes, thereby diminishing inflammatory cell infiltration, aligning with previous findings from probiotic intervention studies [39].

Furthermore, the vaginal microbiota, a vital component of the vaginal barrier, plays a crucial role in maintaining vaginal health [55]. The composition of the vaginal microbial community is primarily characterized by five community state types (CSTs), with four of these CSTs being predominantly composed of a single species of *Lactobacillus* (CST I—*L. crispatus*; CST II—*L. gasseri*; CST III—*L. iners*; CST V—*L. jensenii*), and the fifth being characterized by a dominance of facultative and obligate anaerobes, including *Gardnerella*, *Atopobium*, *Prevotella*, and *Candidatus Lachnocurva Vaginae* [2]. The prevalence of the fifth CST can contribute to vaginal inflammation and disrupt the estrogen-regulated cyclic changes within the vaginal epithelial barrier [54], potentially altering the expression of miRNAs to facilitate the release of E-cadherin, thereby increasing the permeability of the vaginal epithelial barrier [56]. In the present study, infection with *G. vaginalis* increased the relative abundance of *Proteobacteria* and *Actinobacteria* within the vaginal microbiota. However, following intervention with CCFM1339, there was a corresponding increase in the relative abundance of *Firmicutes*, and a rise in the proportion of the *Lactobacillus* genus, which may be associated with the initial modifications to the mechanical and immune barriers. Chen et al. demonstrated that *lactobacillus* intervention could restore the rat vaginal microbiota to normal levels, enhancing the abundance of *Lactobacilli* while reducing the abundance of *Enterobacteriaceae* and *Enterococci* [57]. Similarly, Li et al. manipulated the α-diversity and β-diversity of the vaginal microbiota through a combined microbial intervention approach, successfully reducing the relative abundance of the marker *Escherichia-Shigella* [21].

## 5. Conclusions

The findings revealed that *L. crispatus* CCFM1339 is capable of mitigating the production of biomarkers indicative of vaginal barrier disruption, thereby enhancing the expression of adhesive and desmosomal proteins to reinforce the vaginal mechanical barrier. Concurrently, this strain of Lactobacillus can modulate the expression of anti-inflammatory factors while increasing the levels of proinflammatory factors, thereby inhibiting vaginal inflammation. Moreover, *L. crispatus* CCFM1339 facilitates the expression of vaginal immunoglobulins and defensins, contributing to both the innate and adaptive immune responses, and exerting anti-infective effects. Additionally, this strain promotes the diversity of the vaginal microbiota, increases the abundance of *Firmicutes*, and helps to regulate the microbial ecological balance. This study provides empirical support for the regulatory role of *L. crispatus* CCFM1339 in modulating the vaginal barrier, which may aid in the alleviation of vaginal inflammation.

## Figures and Tables

**Table 1:** Primers used in this study.

Gene	Primer Sequence (5′-3′)
*GAPDH*	Forward: 5′-TGAGTGGCAAAGTGGAGAT-3′
	Reverse: 5′-TTTGCCGTGAGTGGAGTCAT-3′
*IL-17*	Forward: 5′-TGAGTGGCAAAGTGGAGAT-3′
	Reverse: 5′-CTTTCCCTCCGCATTGACAC-3′
*Foxp3*	Forward: 5′-CCCATCCCCAGGAGTCTT-3′
	Reverse: 5′-ACCATGACTAGGGGCACTGTA-3′

## Data Availability

Data used to support the findings of this study are available from the corresponding author upon request.
